# The first cohort of the COVID-19 patients in Vietnam and the national response to the pandemic

**DOI:** 10.7150/ijms.45449

**Published:** 2020-09-09

**Authors:** Ngoc Huy Nguyen, Thong Van Nguyen, An Quang Nguyen, Phuong Van Nguyen, Tuan Ngoc Minh Nguyen

**Affiliations:** 1 General Hospital of Phutho, Vietnam.; 2 Department of Health, Phutho province, Vietnam.; 3 108 Military Central Hospital, Vietnam.

**Keywords:** COVID-19, prevention of disease, controls the outbreak, experience's lesson, Vietnam

## Abstract

The COVID-19 pandemic is a novel infectious disease pandemic with the agent SARS-CoV-2 virus which is currently affecting and causing damage globally. The outbreak has been crossing over 200 countries in the world. In the situation of the outbreak of COVID-19, Vietnam has first sixteen typical cases confirmed positive updated to Feb 28^th^, 2020. After completely applying the medical prevention and active control, Vietnam has the ability to take control of the outbreak of COVID-19 as a recent of WHO assessment. Vietnam has been reported as an effective country for prevention and control the outbreak of COVID-19. We retroactive reviewed our experience with 16 positive cases isolation. This article aims to present the first cohort of COVID-19 patients updated to Feb 28^th^, 2020 in Vietnam and sharing the national response to the pandemic.

## Introduction

On December 31^st^, 2019 the World Health Organization (WHO) has alerted to several cases of a respiratory illness of unknown etiology emerging from Wuhan City, Hubei Province of China 1,9. The clinical presentations resembled viral pneumonia and manifesting as those symptoms of fever, cough and dyspnea 1. As of Jan 30^th^ 2020 the WHO has officially stated this outbreak as a global health emergency. Until April 24^th^ 2020 a total of 2,718,797 confirmed SARS-CoV-2 cases have been reported globally, in which there were 190,656 deaths and 745,813 cases recovered 2.

Currently, over 200 countries and territories have reported cases of COVID-19, typically: America, Spain, Italy, Germany, France, China, United Kingdom, Iran and Turkey 2. These are the countries with the highest number of infections and deaths in the world. Europe is working together with its member states to contain the spread of the virus 3.

Vietnam is among neighboring countries of China sharing a land border of 1,300 kilometers. Both countries keep close long-term bilateral relationship in foreign trade, tourism, diplomacy, science and technology, culture, etc. Furthermore, China has the world's fastest-growing major economy. It is attracting more and more Vietnamese students and employees came to China. Therefore, an epidemic originating from China is unavoidably contagious among people who have been exposed to the virus. Particularly, COVID-19 is novel virus without full understanding until now.

The very first case of COVID-19 in Vietnam was a Chinese man travelling from Wuhan to Hanoi to visit his son living in Vietnam on Jan 13^th^. He was hospitalized on January 22^nd^ at Cho Ray Hospital, Ho Chi Minh City 5, 11. After taking samples and doing all the test of novel corona virus-19, the first case in Vietnam has been confirmed and announced 5.

On February 1^st^ 2020, Prime Minister Nguyen Xuan Phuc signed Decision No.173/QD-TTg announcing the acute respiratory infection epidemic caused by the novel corona virus (nCoV). Accordingly, the Prime Minister announced the outbreak from January 23^rd^ 2020, when the mentioned-before first infected patient was confirmed 5.

On February 3^rd^, Vietnamese government requested completely isolation of immigrants presented in Vietnam who had come to or traveled through 31 epidemic areas in China for previous 14 days, including foreigners. In particular, immigrants who come from or through Hubei Province (China) are considered as cases of illness and must be quarantined right away at local facilities to avoid infection in isolation zone 5.

To actively prevent acute respiratory infections caused by COVID-19 as directed by the Government, provincial authorities throughout the country have taken many measures, namely: cancel events such as festivals, ritual ceremonies, sports tournaments, suspend the operation of businesses activities, amusement park, beauty spas, persuade people not to go out if not needed, keep a distance from others of at least 1 meter in public place, etc. 6. All health facilities, agencies, and unions in the country are fully active to prevent the community spread and combat the epidemic.

WHO has admitted that Vietnam has been doing effectively for the limitation of the outbreak of COVID-19, while there is now having more than 200 emerging countries with COVID-19. Therefore, this article presents the COVID-19 positive cases updated to Feb 28^th^ 2020 in Vietnam and shares the experience in prevention and control the outbreak from Vietnam.

## Materials and Methods

### Data collection process

Information of positive cases was collected from Information Office of Ministry of Health, Vietnam Government, Vietnam and published information to define which medical facility has been in charge of treating patients with COVID-19.

Data then were collected by standardized forms through interviews of infected persons, relatives, close contacts, and health care workers. We collected information on the dates of detection (i.e. the date of positive confirmation of COVID-19), clinical outcomes from health care workers and other information from interviews of infected people.

The strengths of this method are fast and updated. This article is the first synthesis of data on the date of detection, current status, exposure history, specific symptoms and where the patient received treatment until February 28^th^, 2020. However, the weakness of this method is that the trustworthiness still requires further verification.

Regarding to the experiences of Vietnam in prevention and control the outbreak, all information was collected from Ministry of Health of Vietnam via documents namely the Guidance for Diagnosis and treatment for acute respiratory infection caused by SARS-CoV-2 (COVID-19) promulgated together with the decision No.1344/QD-BYT dated March 25^th^, 2020 of Minister of Ministry of Health, official website of Ministry of Health of Vietnam, and the webpage of Ministry of Health of Vietnam on COVID-19.

Information on how Vietnam has responded the pandemic is also found on reports from Ministry of Science and technology of Vietnam, other authors' published articles and world's online journal.

## Results and Discussion

### First 16 positive cases of COVID-19

All of the first 16 confirmed cases infected with the new coronavirus in Vietnam are presented in the following table and synthesized in Appendix 1. They are 16 patients tested positive with nCoV-19 virus by RT-PCR and Next-Generation Gene Sequencing. They were admitted at different hospitals in Vietnam, including: Cho Ray Hospital, Thanh Hoa Hospital, National Hospital for Tropical Diseases, Khanh Hoa Hospital for Tropical Disease, Healthcare Unit of Quang Ha, Tam Dao Healthcare Center 5.

Patients that came from Vinh Phuc are dominant in this first cohort (68.75%). The difference in gender of patient is not significant. In these 16 first cases, there were 8 positive cases that have special symptoms of respiratory travelled from Wuhan and China (Wuhan excluded) and 8 patients were infectious although they had stayed local. All patients had clinical presentations resembling viral pneumonia and manifesting as fever, cough, and dyspnea. (One patient also had diarrhea). And patients had contact to suspected people of COVID-19 infection are dominant (50%). The proportion of infections leading to neurological disease will probably remain small. However, these patients might be left with severe neurological sequelae.

After treatment, all mentioned-above patients have been discharged due to their satisfaction of the following criteria: no fever for at least 3 days, improved clinical symptoms, normal visceral function and blood test result, better chest x-ray result, normal vital signs, at least 2 consecutive negative tests for SARS-CoV-2 (the interval between 2 tests ≥ 24 hour) 11.

### Experiences of Vietnam in prevention and control of the outbreak

Vietnam adopted a quick response under close guidance of the Government, along with the sense of high responsibility of the people, the effectiveness of the political system, especially Ministry of Health as well as other related departments:

#### Government direction and role of stakeholders

The roles in controlling the outbreak of the pandemic are synchronized from the highest to the lowest levels: government, local authorities, and citizens. The rising number of patients has created tremendous community concerns. On January 24^th^ 2020, Vietnam's Civil Aviation Administration directed the cancellation of all flights to and from Wuhan. Operations dependent on human interactions in certain localities were restricted, and initiatives, such as body temperature monitoring, disinfectant use, and the provision of masks, free of charge, in public areas were announced. Controls designed to minimize the infection risks in the city were implemented, such as traffic checkpoints at each province's gateway, closing shops, and establishing disinfection chambers 4.

The Ministry of Education and Training halted all school activities, nationwide, in February and March 2020, as part of quarantine steps to prevent virus transmission. Additionally, the Ministry of Education and Training also altered the time period for national high school examinations, due to the closure of schools 5.

Starting on March 7^th^ 2020, all visitors were expected to declare their health upon entry into Vietnam. On March 11^th^ 2020, Vietnam officially revoked visa exemptions for residents of 8 European countries: Denmark, Norway, Finland, Sweden, the United Kingdom, Germany, France, and Spain and encouraged the use of air-travel masks. On March 14^th^, Vietnam's Ministry of Foreign Affairs announced the Vietnam government's decision to suspend entry for 30 days, starting March 15^th^, 2020, for any citizens traveling from or who have passed through countries, including Schengen, the United Kingdom, and Northern Ireland, within 14 days of their planned arrival date in Vietnam and to temporarily halt the granting of border gate visas. To proactively deter and monitor the dissemination of COVID-19 among the population, starting on March 16^th^ 2020 citizens were required to wear masks in public places 5.

The Ministry of Communications has directed the press agencies to increase the duration, the number of news articles, recommendations, and guide people to be fully aware of risks and how to prevent diseases, raise people's awareness about compliance with recommendations and measures of authorities to limit the spread of epidemics. At the same time, The Ministry of Communications is also directing telecommunication providers to increase coverage on Vietnam's social networks to help people understand and raise awareness about the prevention of COVID-19. The key messages that newspapers and government send to public during this period of time are “stay home”, “wear mask” and “wash hands”. The advice for public is to follow the instructions and regulations during the epidemic such as restricting travel abroad and social distancing. People are required to use personal protective measures such as wearing a face mask and washing their hands regularly when taking public transport or in crowded places. In addition, each citizen has to raise their awareness of protecting the community by reminding family members to follow the rules and instructions. When detecting suspected cases, people should encourage them to go to the nearest medical facilities for timely examination and treatment 6.

#### Vietnam's Covid-19 test kits meet European standards

The test kits were produced by the Vietnam Military Medical University and Viet A company Corporation, which was an assignment given by the Ministry of Science and Technology. On March 15^th^ 2020 at the press conference of the Ministry of Science and Technology, the results of the research on manufacturing Real-time PT-PCR one step biological test kit to detect new strain of corona virus (COVID-19) was announced. Since then, the test kit has been manufactured on a large scale 7.

The British Department of Health and Social Care has granted the CE marking and Certificate of Free Sale (CFS) to Vietnam's test kits used in novel coronavirus disease (COVID-19) diagnosis, according to the Vietnamese government. The simplest testing procedure for COVID-19 is real-time reverse-transcriptase polymerase chain reaction (rRT-PCR). The test can be conducted on either respiratory fluid or blood samples, and typically reports are accessible within a few hours to several days 8.

Screenings, testing using molecular biology techniques on RT-PCR, next-generation gene sequencing machines, confirmation at the Institute of Hygiene and Epidemiology, Pasteur Institute, Centers for Disease Control of provinces and cities have been implemented quickly and precisely. COVID-19 testing process according to the guidance of the Medical Organization World Health (WHO) has been updated regularly contributing to early detection, isolation, treatment of patients 5.

Up to April 27^th^ 2020, there are 112 RT-PCR laboratories nationwide that are capable of detecting SARS-CoV-2 (maximum capacity of ≥ 27.000 samples/day), of which 48 laboratories have been allowed to confirm COVID-19 (maximum capacity is approximately 14.300 samples/day) 7.

#### Treatment

The treatment for COVID-19, which has been implemented following the guidelines of Ministry of Health, Vietnam and WHO, focuses on supportive care to relieve symptoms.

First and foremost, patients are categorized based on the severity of their condition namely suspected cases, mild cases including upper respiratory tract infections or mild pneumonia, severe ones including severe pneumonia or sepsis syndrome and severe critical ones including respiratory distress, ARDS, septic shock or multivisceral failure) 11.

General treatment and follow-up care are the following methods: early functional recovery to improve lung function and others, close monitoring of clinical signs and the progress of lung lesion (EWS can be used to early detect severe signs), supplemental nutrition, cough relief with common drugs or traditional medicines, nose and throat hygiene, warm keeping, adequate water drinking, antipyretic (in case of fever), treatment for accompanied chronic diseases (if any), consultancy, psychological support and encouragement and other treatments including antibiotic, antiviral drugs, corticosteroids, IVIG, interferon, hemodialysis and functional recovery 11.

Used equipments are fingertip pulse oximeter, oxygen respirator, respiratory balloon, electric syringe, etc. for mild cases and HFO ventilator, continuous blood purification, ECMO for severe cases 11.

##### Follow-up after hospital discharge

After being discharged from hospitals, the patients are still kept track. They isolate themselves from others even their family members at home in 14 days after discharge. They have to check their temperature twice a day. If it is over 37.5 °C as of two consecutive times or there are other abnormal signs, they have to have medical check-ups again at medical facilities 11.

Among the patients in the present article, there is no patient that needs medical check-up after being discharged from hospitals 5.

## Conclusion

Vietnam has controlled the outbreak of the pandemic by some reasons. Firstly, the Vietnamese take concern for the pandemic seriously. The second is the synchronization and coordination among government, stakeholders at every level and all citizens. However, vaccination for COVID-19 has been a challenge, since there has been no specific treatment for the disease and the virus still exists. Therefore, the Vietnamese in particular and the world in general still need to stay proactive and alert in the fight against COVID-19.

## Limitations

- Our study is based on positive cases and archived results;
- Our study does not reflect the general population.

## Contributions

All authors contributed equally to the discussions leading to the proposed classification. Tuan Ngoc Minh Nguyen drafted initial and succeeding versions of the manuscript. All authors contributed to the revisions at each stage and all authors read and approved the final manuscript.

## Figures and Tables

**Table 1:** Characteristics of first 16 positive patients of COVID-19 in Vietnam

Characteristics	N	%
**Address**		
Vinh Phuc	11	68.75%
Khanh Hoa	1	6.25%
Ho Chi Minh City	2	12.5%
Thanh Hoa	1	6.25%
Other	1	6.25%
**Gender distribution**		
Male	7	43.75%
Female	9	56.25%
**History of travel**		
Been to Wuhan	7	43.75%
Been to China (Wuhan excluded)	1	6.25%
Stay local	8	50%
**Symptoms**		
Fever	16	100%
Cough	16	100%
Dyspnea	16	100%
Sore throat	0	0%
Vomiting	0	0%
Diarrhea	1	6.25%
Skin hemorrhage	0	0%
Rash	0	0%
**History of exposure**		
Visit any poultry farm/living animal market/contact to animal	0	0%
Contact to suspected people of COVID-19 infection	8	50%
Contact to people back from infectious countries of COVID-19	5	31.25%
Contact to people with symptoms of COVID-19 (cough, fever, dyspnea, pneumonia)	3	18.75%

## Abbreviations

COVID-19: Coronavirus disease 2019
SARS-COV-2: Severe acute respiratory syndrome coronavirus 2
WHO: World Health Organization
RT-PCR: Reverse transcription polymerase chain reaction
NGS: Next-generation sequencing
ARDS: Acute Respiratory Distress Syndrome
EWS: Early Warning Score
HFO: High Frequency Oscillation
ECMO: Extracorporeal Membrane Oxygenation
IVIG: Intravenous immunoglobulin
